# Editorial: New insights in heart valve disease 2022

**DOI:** 10.3389/fcvm.2023.1226113

**Published:** 2023-06-21

**Authors:** Ernesto Greco, Mattia Vinciguerra, Roney Sampaio, Elena Aikawa

**Affiliations:** ^1^ Department of Clinical, Internal Medicine, Anesthesiology and Cardiovascular Sciences, Sapienza University of Rome, Rome, Italy; ^2^ Heart Institute, Clinical Hospital, Faculty of Medicine, University of São Paulo, São Paulo, Brazil; ^3^ Center for Interdisciplinary Cardiovascular Sciences, Brigham and Women’s Hospital, Harvard Medical School, Boston, MA, United States; ^4^ Center for Excellence in Vascular Biology, Brigham and Women’s Hospital, Harvard Medical School, Boston, MA, United States

**Keywords:** valve disease, less invasive approach, phisiopathology, risk stratificacion, guidelines and recommendations

**Editorial on the Research Topic** Insights in heart valve disease: 2022

Heart valve disease (HVD) represents a significant burden for health systems around the world. The growing advances in imaging and diagnostics need to be correlated with improvements in management. In total, nine articles have been published in this Research Topic, with interesting new findings summarized in Table 1. The articles have reached significant visibility, with more than 10,000 views so far. In this editorial, we aim to discuss the articles that highlighted the progress that has been made in the field of heart valve disease in 2022.

**Table 1:** Data regarding the articles published in the research topic “New Insights in Heart Valve Disease”; the number below the authors’ details corresponds to the number of references.

Author	Date of publication	Title	Main findings
Hu et al.	18/10/2022	The projections of global and regional rheumatic heart disease burden from 2020 to 2030	“The RHD burden will remain severe. There are large variations in the trend of RHD burden by region, sex, and age.”
Stöbe et al.	10/01/2023	Left ventricular hypertrophy, diastolic dysfunction and right ventricular load predict the outcome in moderate aortic stenosis	“The presence of two pathophysiological changes is a strong predictor of outcome in moderate AS and may be useful for risk stratification, particularly for scheduling follow-up time intervals and deciding the timing of AVR.”
Han et al.	23/02/2023	Diabetes Is Associated With Rapid Progression of Aortic Stenosis: A Single-Center Retrospective Cohort Study	“Diabetes was strongly and independently associated with rapid progression of AS.”
Salim et al.	07/11/2022	HIF1A inhibitor PX-478 reduces pathological stretch-induced calcification and collagen turnover in the aortic valve	“HIF1A inhibitor PX-478 may impart its anti-calcific and anti-matrix remodeling effect in a stretch independent manner.”
Shechter et al.	02/03/2023	Racial disparities in characteristics and outcomes of patients undergoing mitral transcatheter edge-to-edge repair	“Mitral TEER patients of different racial backgrounds exhibit major differences in baseline characteristics. Among those with functional MR, non-whites and blacks also experience a less favorable 1-year clinical outcome.”
Pugliese et al.	24/03/2023	Flow dynamic assessment of native mitral valve, mitral valve repair, and mitral valve replacement using vector flow mapping intracardiac flow dynamic in mitral valve regurgitation	“Intracardiac flow patterns can be clearly defined using VFM.” Restoration of a physiological blood flow pattern inside the LV directly depends on the procedure used to address MV disease.
Graziani et al.	29/11/2022	Impact of severe valvular heart disease in adult congenital heart disease patients	“In ACHD patients, the presence of S-VHD is independently associated with the occurrence of cardiovascular mortality and hospitalization. The prognostic value of S-VHD is incremental above other established prognostic markers.”
Oettinger et al.	02/05/2023	Treatment of pure aortic regurgitation using surgical or transcatheter aortic valve replacement between 2018 and 2020 in Germany	“TAVR is a viable alternative to SAVR in the treatment of pure aortic regurgitation for selected patients, showing overall low in-hospital mortality and complication rates, especially with regard to self-expanding transfemoral TAVR.”
Yasuhara et al.	28/04/2023	Congenital aortic valve stenosis: from pathophysiology to molecular genetics and the need for novel therapeutics	Narrative review of molecular mechanisms and therapeutic options regarding congenital aortic valve stenosis

## The burden of rheumatic and non-rheumatic valvular disease

The widespread use of penicillin-like drugs and improved access to healthcare reduced the burden of rheumatic heart disease (RHD) in the past century. In some developed countries, the false perception of its under-control burden has diminished alerts in the diagnosis of RHD, worsened by reduced compliance with penicillin treatment. Regional disparities contribute to the vast medical and economic burdens of RHD. It remains the leading cause of severe valvular heart disease with the highest significant cost among cardiovascular diseases, particularly in low-income countries. The valvular involvement frequently leads to heart failure, shortening life expectancy, and is responsible for 320,000 deaths annually. The projections of the global burden in the next decade indicate, mainly in low- and middle-income countries, an increasing trend in incidence, affecting more female subpopulations. The regional disparities may be attributable to the restricted access to healthcare, education, and housing Hu et al.

On the other side of the heart valvular disease spectrum, non-rheumatic, calcific aortic valve and mitral valve diseases are significant causes of public health concern, affecting mainly older adults in Western countries. These are highly treatable diseases and efforts to reduce the burden should be directed to modifiable risk factors, affecting the natural history of the disease, and improving interventional lines of management (1).

## New insights need a new classification

The guidelines suggest treatment for severe valvular disease, identifying echocardiographic parameters of severity that professionals in the medical field can refer to in case of asymptomatic disease. A poor prognosis is associated with severe HVD and its surgical correction is mandatory to reverse cardiac remodeling and restore quality of life.

What emerges from recent literature and most of the articles published in this Research Topic is the necessity to revise surgical timing. The diagnostic assessment needs to include the grading of valve disease, a mirror of the altered morphology, and the dynamic response of the overloaded and/or over-pressured heart chambers. The study of the extra-valve cardiac involvement may allow for defining different stages of the disease, with a notable prognostic impact and crucial role for surgical implications.

## Aortic stenosis

Pathophysiological changes of the left and right ventricles are induced by aortic valve stenosis. Stöbe et al. focused their research on cardiac involvement in patients affected by moderate aortic stenosis. They differentiated the population study according to the number of pathophysiological changes: increased left ventricular mass index, diastolic dysfunction, and increased right ventricle (RV) load. The study population was homogenous for comorbidities, excluding patients with severe comorbidities as these were not attributable entirely to aortic stenosis. The group with more extent cardiac damage resulted in a significantly lower survival rate without aortic valve replacement (AVR) or progression of aortic stenosis. The extra-valve cardiac involvement may be crucial for clinical outcomes after AVR, with a staging classification that appears to have important prognostic implications (2).

The natural history of aortic valve stenosis proceeds along pathophysiological changes and triggering factors may influence the clinical outcomes. A rapid progression of aortic stenosis associated with diabetes was demonstrated by Han et al. with an annual increase of peak aortic jet velocity (V_max_) >0.3 m/s/year. The systemic inflammatory response and impairing endothelial function are recognized as causes of aortic valve degeneration. The adverse effect of diabetes on left ventricle hypertrophic remodeling with increased left ventricle (LV) filling pressures may accelerate the pathophysiological changes, proceeding along with the staging of cardiac damage extent.

The resulting mechanical forces, such as tensile and shear stress, are powerful promoters of aortic valve pathogenesis, with biological responses typical of aortic valve calcification Salim et al. Overall, this emergent staging classification provides an incremental prognostic value in patients with valve disease.

## Mitral regurgitation

The novel classification system was demonstrated to be significantly helpful for risk stratification and timing of surgery for primary mitral regurgitation. Grouping the population study according to the natural history of cardiac involvement in the context of primary mitral regurgitation, for each increase in the group, a 17% higher risk of all-cause mortality was observed (3).

The discussion regarding racial disparities in characteristics and outcomes of patients undergoing mitral transcatheter interventions may lead to full implementation of knowledge of surgical timing Shechter et al. Observations regarding the flow dynamic assessment in the native mitral valve and after surgery may be conducted in a standardized fashion, able to offer valid and crucial results Pugliese et al.

## Conclusion

Identifying factors of greater severity in a patient, with the same group of valvular disease, is fundamental for recognizing the differences in early and long-term prognosis. Although our considerations are focused mainly on surgical timing and prognostic implications, the armamentarium of surgical techniques with less invasive access (4) needs to be at the basis of new studies. While surgical procedures improve the quality of a patient's life, thus far, no drug therapy has been developed to treat HVD, and while efforts have been made to improve surgical techniques and management, further innovative research must be focused on developing non-invasive treatments of valvular diseases.
